# Book Notices

**Published:** 1866-07

**Authors:** 


### EDITORIAL.

### BOOK NOTICES.

*Urinary and Renal Diseases, including Urinary Deposits.* Illustrated by numerous cases and engravings. By William Roberts, M. D., Fellow of the Royal College of Physicians, London; Physician to the Manchester Royal Infirmary; Lecturer on Medicine in the Manchester School of Medicine. Henry C. Lea, Philadelphia. 1866.

One of the most encouraging facts connected with the medical literature of the present time, consists in the originality of the matter which is addressed to the reading public. The day of compilation has passed, and we are seldom obliged to swallow a statement because of the respectable antiquity of the party who first started the tradition. Demonstration is rapidly supplanting hypothesis, and doubt is giving way to the certainty of positive knowledge. The boundary which separates the known from the unknown, is less shadowy and uncertain. Every year sets forth more precisely the exact area of the territory which must yet be explored.

Dr. Roberts has presented the profession with a book, which will be gladly welcomed by all who desire to become acquainted with all that in this year, 1866, is known about urinary and renal diseases. No one can fail to read the work with interest; no physican who is often called to treat cases of urinary and renal diseases, can afford to be without this treatise. Each chapter is a monograph of the particular disease under consideration; and the whole field is thus passed under review in a manner which not only leaves very little to be desired, but also indicates the phenomena that yet demand investigation with a precision which must stimulate the progress of inquiry. The clinical reports of cases are numerous, and are utilized by appropriate commentary, after the example of Bennett in his work on Clinical Medicine. The micrographic illustrations are a whole generation in advance of the delineations of Prout and Golding Bird. From cover to cover, the book is full of meat, and that of the juciest, richest flavor.

For sale by S. C. Griggs & Co., 41 Lake street, Chicago. Price, $4.50.

---

*Biographical Sketches of Distinguished Living New York Surgeons.* By SAMUEL W. FRANCIS, A. M., M. D., etc. New York: John Bradburn. 1866.

The readers of the Philadelphia *Medical and Surgical Reporter* will at once recognize in this volume a reprint of the entertaining sketches which have appeared from time to time in the columns of that periodical. Inheriting his father's social and literary tastes, Dr. Francis has done for his surgical friends that which the celebrated John W. Francis did for the ancient worthies of his own time. Biographical sketches are always read with eagerness — especially when they illustrate those peculiarities which have made men eminent.

The contents of this little volume will be read with avidity by all who have come in contact with the leading surgeons of New York: it supplies precisely that kind of information concerning their preceptors which students like to possess. Only one criticism ;—a little less of euphuistic "sweetness, long drawn out," would have been more agreeable.

---

*Asiatic Cholera.* By F. A. BURRALL, M. D. New York: Wm. Wood & Co., 61 Walker street. 1866.

This little volume will be read with interest by all who desire a clear idea of the history of Cholera. It serves as a panorama by which is displayed all that is really worthy to be preserved from the mass of literature that has grown out of the subject. Having no theory of his own, the author has written in a dispassionate manner, and is content to display only the samples of other men's wares, from which all comers are at liberty to satisfy themselves.

Ticknor and Fields, of Boston, have again laid us under obligations by forwarding to our sanctum their publications for July. The first chapter of Agassiz's forthcoming "Physical History of the Valley of the Amazon" appears in this number of the *Atlantic Monthly*. *Our Young Folks* and *Every Sat-Saturday* are enriched by their usual entertaining miscellany.

This is the species of literature which the late Prof. Chandler Gilman, of blessed memory, was accustomed to recommend to his pupils as appropriate reading for the obstetrical bedside. Certainly, nothing can be imagined better fitted to beguile the tedious hours which so often fall to the lot of the physician.

---

At a recent meeting of the Chicago Medical Society, the members were favored with an exhibition of samples of the different varieties of California wine, introduced by Messrs. Perkins & Stern, of New York. We have always believed in the success of the vineyards of California, and it is now certain that from our Pacific coast are brought wines which are superior to any European wines that can be procured in our market. When we reflect that the medicinal virtues of wine do not merely consist in the fact that the beverage supplies to the patient an agreeable form of dilute alcohol, but rather in the fact that the pure fermented juice of the grape is a complex fluid whose properties are specific, and in their action upon the system analogous to the natural mineral waters, it is easy to see a reason why for the sick room we should prefer an article which, like the California wine, is the genuine product of the vineyard, to the fictitious beverages which are ordinarily sold under the names of port, sherry and champagne. Messrs. Perkins & Stern are the pioneers in the work of introducing the vintage of California to the Atlantic States; and the reputation which they have established, is a sufficient guarantee for the quality of their stock. To the wholesale druggists of this city, they are now furnishing the following wines: port, claret, hock, muscatel and angelica. The port wine is in color and flavor very similar to its European prototype. It contains four per cent. of grape sugar, and 16.5 per cent. of alcohol. In this last particular, it differs greatly from the imported article which, as received from Europe, is always largely reinforced with brandy. The hock is a light, dry wine, free from sugar, and forms, in common with the claret, a very refreshing article for the use of invalids during convalescence from malarial fevers, or in warm weather. The angelica contains fifteen per cent. of alcohol with sixteen per cent. of grape sugar. This constitutes a very nutritious drink, admirably adapted to cases which require the presence of a sugar more assimilable than cane sugar. The muscatel is almost a syrup, containing about six per cent. more sugar than is found in the angelica. One would suppose this wine might be used as an agreeable vehicle for the solution of various drugs.

These wines have been submitted to the most searching tests by the highest medical authorities of the U. S. Army, and have always given perfect satisfaction. We believe that they will soon be used by the whole body of the profession to the utter exclusion of every form of imported wine; and we hope the same may become true of the public at large, for where wine is moderate in price, drunkenness seldom prevails.

---

With the present issue we complete the report of the proceedings of the American Medical Association. For these very full and interesting notes, we are under obligations, many and lasting, to our friends of the *Medical Record.* And by the way, if any of our readers desire to enlarge the circle of their medical reading, they cannot do better than by subscribing for this new semi-weekly serial. For the minutes of the meetings of the Iowa State Medical Society and the Illinois State Medical Association, we are indebted to their Secretaries respectively. We have received full and interesting reports of the annual meetings of many of the County Medical Societies, which have been crowded out for want of space on our pages. It is gratifying to remark the enthusiasm with which the profession has resumed the appropriate arts of peace; and in no quarter are these manifestations more satisfactory than in the Southern States. In addition to the Journals published at Richmond, Savannah, Atlanta and Memphis, which we have already noticed, we can now welcome the "Southern Journal of the

Medical Sciences," published in New Orleans by an association of physicians, at the head of whom stood the late Dr. E. D. Fenner, whose death occurred in April, almost simultaneously with the appearance of the new journal to whose establishment he had so largely contributed. The *New Orleans Medical Record* is a semi-monthly devoted to medical science, under the editorial care of the celebrated Dr. Bennet Dowler. These new journals are excellent, and promise well for the progress of true medical science in that semi-tropical zone upon the shores of the Mexican Gulf.

---

As the season of vile odors and foul miasms is opening upon us with a dark cloud of cholera glooming in the horizon, the question of disinfectants assumes more than ordinary interest. We notice in the daily papers a circular from Dr. S. C. Blake, tne city physician, recommending the use of chloride of zinc for the purification of basements, vaults and sick rooms. In this he agrees with the distinguished chemist, Prof. Blaney, of Rush Medical College, who, in a recent letter to Mr. E. H. Sargent, the manufacturer, writes as follows: "There is abundant scientific authority—with which my own experience accords—to prove beyond doubt the immense value of this agent as a deodorizer, and, in a high degree, as a disinfectant. While it is equally as prompt and efficient as chloride of lime, the best known and the longest used of the deodorizers, it has over it the great advantage that it has no offensive odor of its own, when neutral, and may be freely used about the persons and clothing of the most sensitive persons without exciting any feeling of disgust. As a deodorizer, for use in closed apartments, and within dwellings for general family use, I am of the opinion that the chloride of zinc is the most SAFE, the most PROMPT, and the most EFFICIENT of the deodorizers now furnished for general distribution."

---

Special attention is requested to the advertisement of the Prize Committee of the Indiana State Medical Society, which will be found in our advertising sheet.

